# A Cross-Tissue Transcriptome-Wide Association Study Identifies Novel Susceptibility Genes for Glomerular Diseases

**DOI:** 10.3390/biomedicines14051072

**Published:** 2026-05-08

**Authors:** Lichao Mao, Linhong Xu, Tong Zhu, Xintong Liu, Zehua Li

**Affiliations:** 1 Renal Division, Peking University Institute of Nephrology, Peking University First Hospital, Beijing 100034, China; lichaomao1022@gmail.com (L.M.); 2210301155@stu.pku.edu.cn (L.X.); 2310301156@stu.pku.edu.cn (T.Z.); 2010301136@stu.pku.edu.cn (X.L.); 2 Key Laboratory of Renal Disease-Ministry of Health of China, Key Laboratory of Chronic Kidney Disease Prevention and Treatment (Peking University)-Ministry of Education of China, Peking University First Hospital, Beijing 100034, China; 3 Research Units of Diagnosis and Treatment of Immune-Mediated Kidney Diseases, Chinese Academy of Medical Sciences, Peking University First Hospital, Beijing 100034, China; 4 Beijing Key Laboratory of Precision Medicine and New-Drug/Equipment Development for Severe Kidney Disease, Beijing 100034, China

**Keywords:** glomerular disease, cross-tissue transcriptome-wide association study, novel susceptibility genes, druggable target, Mendelian randomization

## Abstract

**Background/Objectives**: Glomerular diseases (GD) possess strong polygenic susceptibility, yet exact causal genes remain unclear because most variants identified by genome-wide association studies (GWAS) reside in non-coding regions. While transcriptome-wide association studies (TWAS) effectively decode complex traits, cross-tissue profiling for GD remains largely unexplored. Therefore, this study employs an integrative cross-tissue TWAS and Mendelian randomization framework to systematically identify and validate novel GD susceptibility genes. **Methods**: We conducted a systematic cross-tissue TWAS integrating Genotype-Tissue Expression (GTEx) v8 eQTL data across 49 tissues. Candidate genes were nominated using five complementary frameworks (sparse canonical correlation analysis (sCCA), functional summary-based imputation (FUSION), fine-mapping of causal gene sets (FOCUS), summary-data-based Mendelian randomization (SMR), and multi-marker analysis of genomic annotation (MAGMA)). Findings were refined via Mendelian randomization (MR), pathway enrichment, protein interaction networks, and druggability profiling. **Results**: We identified 21 candidate susceptibility genes for GD, with 10 genes (*AGER*, *C6orf48*, *CSNK2B*, *CYP21A2*, *HLA-DRB1*, *HSD17B8*, *LST1*, *MICB*, *PRRT1*, *TCF19*) strongly supported by MR analysis. Notably, five of these MR-prioritized genes (*C6orf48*, *CSNK2B*, *HSD17B8*, *LST1*, and *PRRT1*) were previously unreported. Functionally, these prioritized genes are primarily involved in immune modulation, inflammation, and steroid metabolism. Furthermore, five genes (*AGER*, *CSNK2B*, *CYP21A2*, *HLA-DRB1* and *MICB*) were identified as potentially druggable targets. **Conclusions**: This first systematic cross-tissue TWAS of GD prioritizes a set of genetically supported susceptibility genes. By uncovering novel drivers and druggable proteins, this study advances the mechanistic understanding of GD and provides a foundation for future therapeutic development and precision nephrology.

## 1. Introduction

Glomerular disease (GD) is a leading cause of chronic kidney disease (CKD) and end-stage renal disease (ESRD), imposing a substantial global health burden [1,2,3,4]. Beyond primary localized injury to the glomeruli, GD is increasingly recognized as a complex systemic disorder driven by widespread multi-organ crosstalk [5,6]. This systemic complexity is clinically mirrored by the significant global variation and challenging management of major subtypes, including IgA nephropathy (IgAN), focal segmental glomerulosclerosis (FSGS), and membranous nephropathy (MN) [7,8,9,10]. Current untargeted therapies often yield variable responses, highlighting a critical need to decode the underlying molecular pathogenesis of GD [1,2,3,4,10]. Crucially, GD pathogenesis is fundamentally governed by genetic susceptibility. While rare early-onset cases are monogenic, the vast majority of adult-onset GD exhibits a highly polygenic architecture driven by the cumulative effects of common single-nucleotide polymorphisms (SNPs) [5,7,11]. Deciphering this polygenic complexity via genome-wide approaches is imperative to unravel the molecular drivers of GD and advance precision medicine [8,12].

Genome-wide association studies (GWAS) serve as the gold standard for deconstructing the polygenic architecture of complex traits [13]. In GD, GWAS have mapped critical susceptibility loci, including *HLA* variants for IgAN [8,12] and MN [14], and *APOL1* for FSGS [15]. However, pinpointing exact causal genes remains challenging, as most identified variants localize to uncharacterized non-coding regions [16]. Furthermore, structural complexities introduced by linkage disequilibrium (LD) and distal regulatory elements (e.g., long-range enhancers) dictate that the gene physically proximal to a lead SNP is frequently not the true biological driver [17,18].

Transcriptome-wide association studies (TWAS) overcome GWAS limitations by integrating expression quantitative trait loci (eQTL) data to prioritize candidate genes [19,20], thereby enhancing statistical sensitivity and minimizing spurious associations [21]. A variety of TWAS-centric analytical paradigms have emerged, encompassing functional summary-based imputation (FUSION) [19], fine-mapping of causal gene sets (FOCUS) [22], and summary-data-based Mendelian randomization (SMR) [23], each offering unique statistical strategies for prioritizing susceptibility genes. Coupled with Mendelian randomization (MR) for validation, TWAS mitigate confounding from linkage disequilibrium or pleiotropy [24]. Furthermore, leveraging multi-tissue datasets like GTEx v8 allows TWAS to capture the tissue-specific and systemic regulatory patterns critical to GD’s heterogeneous cellular landscape [25,26]. The synergistic integration of multi-tissue and tissue-specific TWAS methodologies enables the cross-validation of genetic signals and the detection of genes with pervasive pleiotropic effects, ultimately yielding a more holistic understanding of the regulatory landscape underlying disease development. This unified framework has successfully unmasked novel susceptibility genes across complex traits, including Alzheimer’s disease (AD) [25], migraine [27], coronary artery disease [28], and ulcerative colitis [29].

While cross-tissue TWAS and Mendelian randomization (MR) frameworks have demonstrated profound potential in deciphering complex renal traits—including CKD and our group’s recent application to diabetic kidney disease (DKD) [20,30,31,32]—a comprehensive systemic identification of susceptibility genes for GD remains elusive. Current genetic studies on GD subtypes are predominantly confined to single-tissue renal analyses, neglecting critical systemic regulatory networks [8,33,34]. This is a major limitation, as GD pathogenesis often originates extra-renally; for instance, IgAN is driven by anomalous mucosal immunity [35], with susceptibility loci heavily enriched in systemic immune and intestinal networks rather than isolated renal compartments [12]. To bridge this gap, we applied an integrative cross-tissue TWAS and MR approach across 49 tissues. We identified and prioritized ten GD susceptibility genes (*HSD17B8*, *TCF19*, *AGER*, *CYP21A2*, *PRRT1*, *C6orf48*, *LST1*, *HLA-DRB1*, *CSNK2B*, and *MICB*) predominantly implicated in systemic immune activation, metabolism, and cell-intrinsic regulatory. Ultimately, these findings decode the systemic genetic architecture of GD, laying a robust foundation for future targeted therapeutics.

## 2. Materials and Methods

### 2.1. Study Design

**Figure 1:**
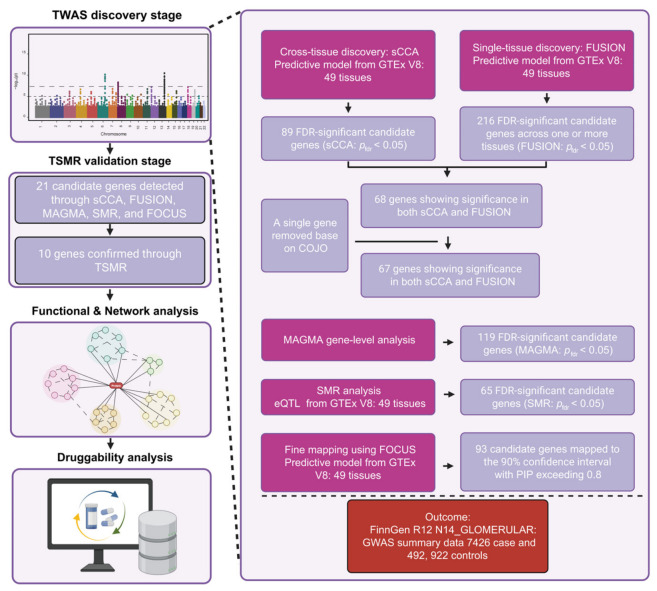
Systematic workflow of multi-stage exploratory transcriptome-wide association study (TWAS) analysis in glomerular disease (GD). An overview of the multi-tier analytical pipeline designed to integrate various TWAS strategies for GD susceptibility gene identification. The process begins at the discovery stage, leveraging genome-wide association study (GWAS) summary statistics from the FinnGen R12 cohort (7426 cases and 492,922 controls) in conjunction with expression quantitative trait loci (eQTL) data across 49 human tissues from the Genotype-Tissue Expression (GTEx) v8 database. Five complementary analytical frameworks—comprising summary-data-based Mendelian randomization (SMR), multi-marker analysis of genomic annotation (MAGMA), functional summary-based imputation (FUSION), sparse canonical correlation analysis (sCCA), and fine-mapping of causal gene sets (FOCUS)—were concurrently applied to nominate distinct sets of candidate genes. To ensure independent signals, the conditional and joint association analysis (COJO) was applied to refine single-tissue candidates, resulting in the removal of specific genes before the final overlap analysis with joint tissue findings. During the validation stage, Two-Sample Mendelian Randomization (TSMR) analysis was performed using external data to refine the selection, ultimately identifying 10 prioritized susceptibility genes for GD. To characterize the biological roles of these genes, functional enrichment and gene network analyses were conducted. Lastly, druggability profiling was carried out to assess the viability of these genes as prospective therapeutic targets. The colors of the boxes provide a functional legend: magenta boxes represent broad analytical frameworks; lavender boxes indicate specific processing details, candidate gene counts, and the COJO filtering step; and the coral box specifies the GWAS outcome data source. The dashed lines delineate key areas of the workflow: two lines connect the diagram of the discovery stage to the detailed flowchart; and another line separates the functional verification steps from the final GWAS source data details. Created in BioRender. Li, Z. (2026) https://BioRender.com/yq167nj (accessed on 29 April 2026).

The overall study design is summarized in Figure 1. We conducted a cross-tissue TWAS integrated with two-sample MR to identify and validate susceptibility genes for GD. The analytical pipeline included: (1) cross-tissue TWAS using sparse canonical correlation analysis (sCCA) to detect genetic effects shared among multiple tissues, (2) single-tissue TWAS using FUSION to identify genes overlapping with sCCA-selected candidates, (3) conditional and joint (COJO) analysis to account for false-positive signals arising from LD, (4) gene-level analysis with multi-marker analysis of genomic annotation (MAGMA), (5) pleiotropic association analysis via SMR, (6) fine-mapping with FOCUS, and (7) two-sample MR analysis to further validate the identified genes and evaluate genetic associations. Gene network analysis and functional and pathway enrichment analyses were implemented via Gene Multiple Association Network Integration Algorithm (GeneMANIA), the STRING database, and Metascape to uncover underlying biological connections; subsequently, druggability analysis was conducted to evaluate the feasibility of these genes as therapeutic targets.

### 2.2. GWAS Summary Statistics

GWAS summary data for GD were derived from the FinnGen R12 cohort (https://www.finngen.fi/en, accessed on 8 December 2024), using the N14_GLOMERULAR endpoint, which encompasses ICD-10 codes for glomerular diseases (e.g., N00-N08, including IgA nephropathy, focal segmental glomerulosclerosis, and membranous nephropathy). The dataset comprised 7426 cases and 492,922 controls of European ancestry.

### 2.3. eQTL Reference Panel

The eQTL data were extracted from the GTEx project v8 database, which encompasses comprehensive gene expression landscapes across 49 human tissues from a cohort of 838 post-mortem donors. This dataset was retrieved from the European Bioinformatics Institute (EBI) repository (Hinxton, UK) (https://ftp.ebi.ac.uk/pub/databases/spot/eQTL/imported/GTEx_V8, accessed on 8 December 2024).

### 2.4. Cross-Tissue TWAS Using sCCA

We implemented a cross-tissue TWAS framework utilizing sCCA, predicated on GTEx v8 multi-tissue eQTL panels. By identifying a sparse linear combination of tissue-specific expression levels, sCCA optimizes the correlation with cis-genetic variants to integrate gene expression across various tissues. The resulting canonical loadings quantify the contribution of each tissue to the predictor, facilitating feature selection while maximizing the genotype–expression association—a method that offers superior performance over traditional principal components analysis (PCA) or weighted averaging approaches [36]. Association testing was conducted between sCCA-derived predicted expression and GD GWAS statistics, with the findings subsequently integrated with single-tissue TWAS results via the Aggregate Cauchy Association Test (ACAT). All computational analyses were restricted to autosomal chromosomes using default parameters, with significance determined by an FDR-adjusted threshold (p<0.05). LD patterns were estimated from the 1000 Genomes Project (Phase 3) European reference panel.

### 2.5. Single-Tissue TWAS Using FUSION

We implemented the FUSION toolkits (http://gusevlab.org/projects/fusion/, accessed on 10 December 2024) to evaluate candidate genes for GD by leveraging GTEx V8 multi-tissue eQTL weights and GWAS results. The analysis began with LD estimation between SNPs and predictive models using European samples from the 1000 Genomes Project. To model the genetic architecture of gene expression, FUSION utilizes five algorithms (best linear unbiased prediction (BLUP), Bayesian sparse linear mixed model (BSLMM), least absolute shrinkage and selection operator (LASSO), Elastic Net, and top-1) and selects the most robust performer based on predictive accuracy. The resulting weights were then cross-referenced with GD GWAS-derived *Z*-scores to infer gene–trait associations [37]. Genes were considered significantly associated if they achieved a false discovery rate (FDR)-adjusted *p*-value < 0.05 in one or more tissues.

### 2.6. Conditional and Joint Analysis

To discern independent genetic signals from multiple associated features within a single locus, we employed COJO analysis, a post-processing module within FUSION [19]. By explicitly accounting for LD between markers, COJO facilitates a more nuanced understanding of the genetic architecture underlying the trait [38]. Following these tests, genes that retained their associations were classified as jointly significant, whereas those whose significance was attenuated after adjustment were defined as marginally significant.

### 2.7. MAGMA Gene-Level Analysis

Gene-level associations were estimated using MAGMA (version 1.08; Department of Complex Trait Genetics, Center for Neurogenomics and Cognitive Research, VU University Amsterdam, Amsterdam, The Netherlands; Institute for Computing and Information Sciences, Radboud University Nijmegen, Nijmegen, The Netherlands) under default settings, which consolidates individual SNP association statistics into unified gene scores. This procedure allows for the quantification of the genetic contribution of each gene to the studied phenotype [39]. For a comprehensive overview of the methodological framework and specific parameter configurations, we refer the reader to the official MAGMA documentation.

### 2.8. SMR Analysis

To refine and substantiate the candidates identified via TWAS, we performed SMR using the official SMR software package (version 1.3.1; Westlake University, Hangzhou, China; https://yanglab.westlake.edu.cn/software/smr, accessed on 10 December 2024) [23]. This method was employed to integrate GD GWAS statistics with GTEx V8 eQTL data across 49 tissues within a linear regression framework. To distinguish shared genetic effects (genuine pleiotropy) from confounding due to genetic linkage, we implemented the heterogeneity in dependent instruments (HEIDI) test. Instrumental SNPs exhibiting significant heterogeneity (unadjusted HEIDI *p*-value ≤ 0.01) were excluded to ensure the reliability of prioritized genes. In the multi-tissue SMR analysis, significance was defined as a *p*-value < 0.05 in at least one tissue.

### 2.9. Fine-Mapping Using FOCUS

To conduct fine-mapping of TWAS-associated genomic risk regions, we utilized FOCUS (pyfocus version 0.802) to perform fine-mapping and identify potentially susceptibility genes. This tool facilitates the elucidation of genomic risk by integrating GWAS summary statistics with GTEx V8 eQTL weights (spanning 49 tissues) to generate a credible set of genes [22]. For our GD analysis, risk genes were prioritized based on stringent criteria: (1) a posterior inclusion probability (PIP) > 0.8; (2) a significance threshold of $p<5\times 10^{-8}$; and (3) inclusion within the 90% credible intervals (CIs).

### 2.10. Two-Sample MR

To evaluate genetically predicted associations, we implemented a two-sample MR analysis using the TwoSampleMR package (version 0.6.6; MRC Integrative Epidemiology Unit, University of Bristol, Bristol, UK). Independent cis-eQTL SNPs were leveraged as instrumental variables (IVs), with gene expression defined as the exposure and GD as the clinical outcome. We first ascertained genome-wide significant variants ($p<5\times 10^{-8}$) and applied LD clumping ($r^{2}<0.001$) to ensure the independence of genetic proxies [40]. Given that each gene was represented by a single independent instrument, MR effect estimates were calculated via the Wald ratio method, with statistical significance determined at p<0.05.

### 2.11. Functional Enrichment Analysis and Gene Network Analysis

Functional enrichment analysis of the ten susceptibility genes was conducted using the Metascape platform (http://metascape.org, accessed on 10 January 2026) [41]. A *p*-value threshold of <0.05 was considered statistically significant for enriched terms. The primary objective of this analysis was to identify significantly overrepresented biological processes and molecular pathways among the candidate genes.

To facilitate the functional annotation and interaction profiling of the prioritized genes, we utilized GeneMANIA (http://genemania.org, accessed on 26 February 2026) alongside the STRING database (https://string-db.org, accessed on 26 February 2026). While GeneMANIA serves as a multifaceted resource that synthesizes diverse biological data—encompassing co-expression patterns, molecular pathways, and genetic interactions—to predict functional connectivity, STRING was specifically employed to construct comprehensive protein–protein interaction (PPI) networks. Together, the integration of these platforms delineates both the broad functional relationships and the direct physical interactions underlying the biological mechanisms of the candidate gene set.

### 2.12. Druggability Assessment

To evaluate the therapeutic tractability of MR-validated genes, we cross-referenced our candidates with the druggable genome (comprising 4479 genes) as defined by Finan et al. [42]. These genes were stratified into three tiers based on their status in the drug development pipeline. Tier 1 (1427 genes) includes efficacy targets for approved drugs or candidates currently in clinical trials. Tier 2 (682 genes) consists of proteins with known bioactive small-molecule binders or those exhibiting ≥ 50% sequence identity to approved targets. Tier 3 (2370 genes) encompasses targets with more distal homology to approved drugs or members of recognized druggable families not captured in Tiers 1 or 2. Furthermore, the clinical development phases and corresponding pharmacological agents were annotated using the therapeutic target database (TTD) (http://db.idrblab.net/ttd/, accessed on 5 January 2026).

To overcome the inherent lag of static databases and comprehensively evaluate the translational feasibility of the prioritized targets in GD, we subsequently expanded this analytical framework. Real-time clinical trial progressions, phase advancements, and novel indications for specific agents were systematically curated from the ClinicalTrials.gov registry. Furthermore, specific drug-target interactions and the availability of pharmacological modulators were cross-verified utilizing the Small Molecule Suite (https://labsyspharm.shinyapps.io/smallmoleculesuite/, accessed on 14 April 2026). Finally, a targeted literature review was conducted to contextualize the biological functions, spatial expression patterns, and potential mechanistic contraindications of these pharmacological interventions within the specific microenvironment of glomerular diseases.

### 2.13. Statistical Analysis

FDR adjustments used the Benjamini–Hochberg method. The positive space for each method was defined separately in the preceding sections.

### 2.14. Ethical Considerations

This study used publicly available, de-identified data, requiring no institutional review board approval.

## 3. Results

### 3.1. Discovery of GD Susceptibility Genes Through Integrative TWAS Analysis

To identify susceptibility genes for GD (FinnGen endpoint N14_GLOMERULAR), we integrated GTEx v8 eQTL data (49 tissues) with GWAS summary statistics from the FinnGen R12 cohort, including 7426 cases and 492,922 controls (Figure 1). Cross-tissue TWAS using sCCA detected 89 genes with significant expression–trait associations (FDR<0.05) (Supplementary Tables S1 and S2). Single-tissue TWAS, conducted via FUSION across 49 tissues, identified 216 genes, including 68 shared with sCCA and 148 unique to FUSION (Supplementary Figure S1 and Tables S3 and S4). To ensure independent associations, we applied COJO analysis. Specifically, NFKBIL1 was excluded from the final joint model (Supplementary Table S5) because its conditional *p*-value failed to reach the significance threshold after conditioning on the lead signals. This exclusion is indicative of the high degree of linkage disequilibrium (LD) architecture at this locus, representing a shared genetic signal rather than a failure of colocalization. The remaining candidate genes were further prioritized using several complementary TWAS methods. MAGMA gene-level analysis, aggregating SNP signals within ±10 kb of gene boundaries, nominated 119 genes (FDR<0.05) (Supplementary Table S6). MAGMA gene-set analysis (GSA) revealed a significant enrichment in major histocompatibility complex (MHC) Class II pathways, complemented by prominent gene-level signals at the *HLA-DQA1*, *PRRT1*, *TNXB*, *CLIC1*, *MSH5*, and *CYP21A2* loci (Supplementary Figure S2A). These signals spanned multiple systemic tissues, suggesting that multi-organ inflammatory genes mediate the genetic susceptibility to GD (Supplementary Figure S2B). SMR analysis further prioritized 65 genes with pleiotropic associations (SMR $p<5\times 10^{-5}$, HEIDI p>0.01 to exclude pleiotropy) (Supplementary Table S7). To refine the genetic signals, we performed fine-mapping with FOCUS and identified 93 genes with a PIP>0.8, confirming their likely genetic associations with GD (Supplementary Table S8).

**Figure 2:**
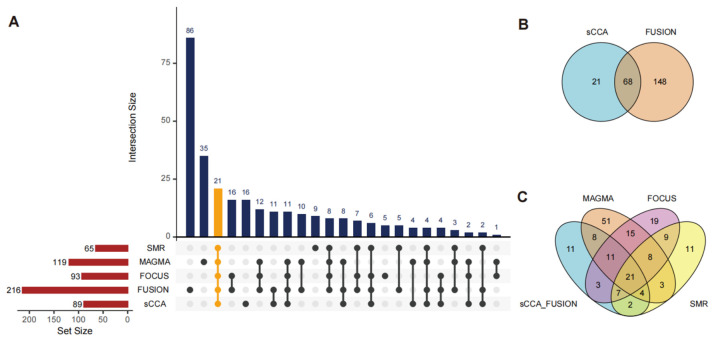
Multi-method integration of TWAS approaches in GD. Overlapping genes identified through various analytical frameworks are presented via an intersection plot (**A**) and Venn diagrams (**B**,**C**). Within the intersection plot (**A**), the top panel illustrates the sizes of various intersections, where the height of each bar corresponds to the number of overlapping genes. The height of each dark blue bar corresponds to the number of genes in that unique subset. The bottom panel indicates the total gene set size for each individual method using red bars. Specific combinations of these methods are denoted by the matrix of black dots and connecting lines. FUSION, MAGMA, FOCUS, sCCA, and SMR identified 216, 119, 93, 89, and 65 candidate genes, respectively. The 21 genes consistently detected by all five analytical methods are highlighted in orange. Abbreviations: SMR: Summary-data-based Mendelian randomization; MAGMA: Multi-marker analysis of genomic annotation; FOCUS: Fine-mapping of causal gene sets; FUSION: Functional summary-based imputation; sCCA: Sparse canonical correlation analysis.

By cross-referencing these approaches, we identified 21 high-confidence candidate genes, each supported by all five methods, including MHC genes (*HLA-DQB1*, *HLA-DRB1*, *HLA-DQA1*, *HLA-DMA*, *HLA-DRA*), alongside 16 non-MHC genes (*TCF19*, *TAP1*, *HSD17B8*, *AGER*, *PRRT1*, *SKIV2L*, *NELFE*, *EHMT2*, *C6orf48* (also known as *SNHG32*), *MSH5*, *CSNK2B*, *APOM*, *LST1*, *MICB*, *DDAH2*, and *CYP21A2*) (Figure 2A–C). These genes were advanced for further validation, reflecting robust associations with GD risk across systemic and tissue-specific contexts.

### 3.2. Validation of Genetic Associations via Two-Sample MR

**Figure 3:**
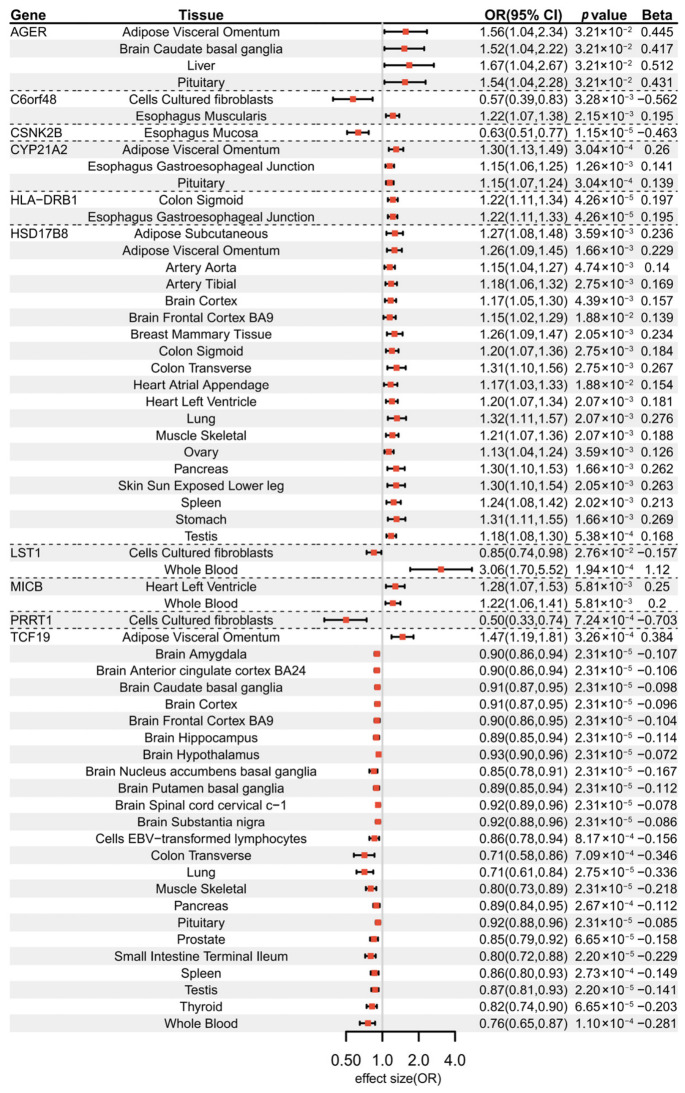
Multi-method, tissue-specific genetic effects of GD-associated genes. Forest plots showing odds ratios (ORs) and 95% confidence intervals (CIs) for various gene-tissue pairs identified through MR analysis. Effect sizes are displayed on the log scale, where OR>1 indicates risk-increasing and OR<1 indicates protective effects. Significant associations (p<0.05) are presented for the 10 validated susceptibility genes (*AGER*, *C6orf48*, *CSNK2B*, *CYP21A2*, *HLA-DRB1*, *HSD17B8*, *LST1*, *MICB*, *PRRT1*, and *TCF19*) across diverse human tissues.

Two-sample MR, using eQTLs as instrumental variables, supported significant associations between gene expression and GD risk. Across 49 tissues, 10 genes showed significant MR associations (p<0.05) (Supplementary Table S9). These genes can be classified into three categories based on their risk and protective effects across different tissues. Five genes exhibited a consistent risk effect on GD across all tissues, including *AGER* (odds ratios (OR) ranging from 1.52 to 1.67, $p=3.01\times 10^{-2}$), *CYP21A2* (OR ranging from 1.15 to 1.30, *p* values from $3.04\;\times \;10^{-4}$ to $1.26\times 10^{-3}$), *HLA-DRB1* (OR: 1.22, 95% CI: 1.11–1.34, $p=4.26\times 10^{-5}$), *HSD17B8* (OR ranging from 1.13 to 1.32, *p* value from $5.38\times 10^{-4}$ to $1.88\times 10^{-2}$), *MICB* (OR ranging from 1.22 to 1.28, $p=5.81\times 10^{-3}$). Genes exhibiting exclusively protective effects included *CSNK2B* (OR: 0.63, 95% CI: 0.51–0.77, $p=1.15\times 10^{-5}$) and *PRRT1* (OR: 0.50, 95% CI: 0.33–0.74, $p=7.24\times 10^{-4}$). Three genes showed bidirectional associations across tissues. Specifically, *C6orf48* showed a positive association in the esophagus muscularis (OR: 1.22, 95% CI: 1.07–1.38, $p=2.15\times 10^{-3}$), but an inverse association in cultured fibroblasts (OR: 0.57, 95% CI: 0.35–0.83, $p=3.28\times 10^{-3}$). *LST1* showed a protective association in cultured fibroblasts (OR: 0.85, 95% CI: 0.74–0.98, $p=2.76\times 10^{-2}$), while positively associated with GD in the whole blood (OR: 3.06, 95% CI: 1.70–5.52, $p=1.94\times 10^{-4}$). In addition, *TCF19* showed inverse associations across most tissues (OR ranging from 0.71 to 0.93, *p* values from $2.20\times 10^{-5}$ to $8.17\times 10^{-4}$) except in the adipose visceral omentum (OR: 1.47, 95% CI: 1.19–1.81, $p=3.26\times 10^{-4}$) (Figure 3). Among all the genes showing significant effects, five of them have not been previously implicated in direct association with GD in systematic searches of PubMed, GWAS Catalog [43], Open Targets [44], and ClinVar [45] databases: *HSD17B8*, *PRRT1*, *C6orf48*, *LST1*, and *CSNK2B*.

### 3.3. Functional Enrichment and Network Analysis

Functional enrichment analysis of the 10 susceptibility genes was performed using the Metascape platform. This analysis identified two primary biological clusters and several specific molecular pathways. The most significant enrichment was observed in the regulation of lymphocyte proliferation (p<0.0001), primarily driven by *AGER*, *HLA-DRB1*, *LST1*, and *CSNK2B* (Supplementary Figure S3). Additionally, significant enrichment was found in the regulation of hormone levels (p<0.001) and the metabolism of lipids (p<0.01), involving *CYP21A2*, *HLA-DRB1*, *HSD17B8*, and *CSNK2B* (Supplementary Figure S3). At the gene-specific level, *HLA-DRB1* is associated with MHC class II antigen processing and the interferon-gamma response, while *CYP21A2* and *HSD17B8* are involved in steroid hormone biosynthetic processes. Furthermore, *CSNK2B* is linked to PI3K/AKT/mTOR signaling, *PRRT1* to α-amino-3-hydroxy-5-methyl-4-isoxazolepropionic acid receptor activity and synaptic depression, and *TCF19* to the regulation of gene expression and E2F targets (Supplementary Table S10).

**Figure 4:**
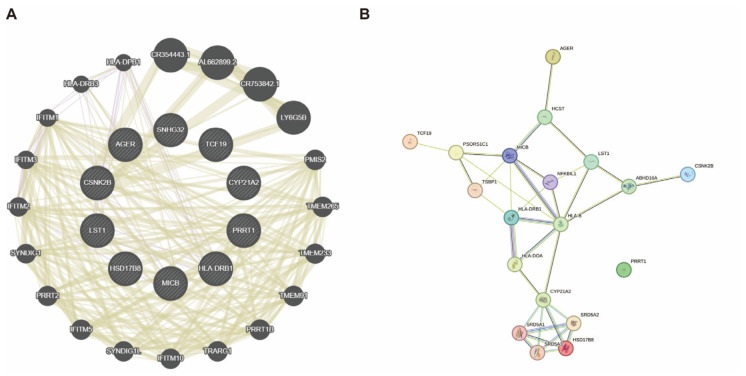
Interaction network analyses of prioritized GD-associated candidate genes. (**A**) GeneMANIA gene network. The size of each node correlates with the gene’s significance in the TWAS analysis, with these input candidate genes indicated by striped shading. The edges represent various types of gene–gene interactions. Edge colors denote the type of evidence: pink/purple lines represent co-expression, tan lines indicate co-localization, and other muted colors represent alternative categories. (**B**) Protein–protein interaction (PPI) network generated via the STRING database. Nodes represent all proteins produced by a single protein-coding gene locus. Colored nodes indicate query proteins and the first shell of interactors, while white nodes represent the second shell of interactors; filled nodes indicate a known or predicted 3D protein structure. Edges represent specific and meaningful protein–protein associations (joint contributions to a shared function, rather than strictly physical binding). Edge colors denote the type of supporting evidence: cyan (curated databases) and purple (experimentally determined) for known interactions; green (gene neighborhood), red (gene fusions), and dark blue (gene co-occurrence) for predicted interactions; and light green (text mining), black (co-expression), and light blue (protein homology) for other associations.

To elucidate the underlying biological mechanisms and functional relationships among the prioritized GD-associated candidate genes, we constructed interaction networks utilizing GeneMANIA and the STRING database (Figure 4). The GeneMANIA network (Figure 4A) revealed a highly interconnected functional architecture among the candidate genes. Functional enrichment within this expanded network demonstrated a strong convergence on immune-related activities, specifically highlighting MHC class II receptor activity, peptide antigen binding, and the regulation of immune response signaling pathways. Complementing the broader functional network, the STRING PPI analysis (Figure 4B) delineated specific physical and functional associations at the protein level. The PPI network exhibited distinct topological clustering. A prominent immune-centric cluster was organized around prioritized genes HLA-DRB1 and MICB, along with their primary interactors such as HLA-B and HLA-DOA, underscoring their closely coordinated roles in antigen processing and systemic immune presentation. Concurrently, a distinct metabolic cluster emerged at the bottom of the network, predominantly involving CYP21A2 and HSD17B8 (interacting with the SRD5A protein family), which pointed towards steroid hormone biosynthesis and metabolic regulation. Collectively, these multi-layered network analyses indicate that the identified susceptibility genes operate through a coordinated systemic network, primarily driving GD pathogenesis via coupled immune dysregulation and metabolic alterations.

### 3.4. Druggability and Expression Profiles

To determine the therapeutic potential of the MR-validated genes, we evaluated whether their encoded proteins could function as viable drug targets. By cross-referencing our findings with the dataset established by Finan et al. and the TTD, we identified three of the ten candidate genes as encoding proteins with established druggability [42]. Specifically, HLA-DRB1 is categorized as a Tier 1 druggable target, for which glatiramer acetate is an approved pharmacological agent. In contrast, AGER and CYP21A2 are designated as Tier 3 targets; their respective candidates, PF-4494700 and BBP-631, are currently in clinical development (Table 1). Although AGER is formally classified as a Tier 3 target within this historical framework, our updated real-time investigation highlights its rapidly evolving therapeutic potential. Specifically, its antagonist Azeliragon (also known as PF-4494700) has progressed to Phase 3 clinical trials (NCT05815485) and has previously demonstrated clinical efficacy in reducing the urinary albumin-to-creatinine ratio in a Phase 2a trial for diabetic nephropathy (NCT00287183). Furthermore, while the novel targets MICB and CSNK2B were not initially captured within the static Finan et al. dataset, our expanded analytical framework revealed their emerging therapeutic tractability [42]. Targeted curation of the Therapeutic Target Database and ClinicalTrials.gov registry demonstrated that both targets are actively being investigated in clinical trials (Table 1). Collectively, these findings underscore the high translational priority of these candidates in the context of glomerular injury and highlight the critical need to supplement static genomic frameworks with real-time clinical progression data.

## 4. Discussion

GD are a major cause of end-stage renal disease and cardiovascular mortality worldwide, yet their genetic architecture remains incompletely understood. By integrating cross-tissue transcriptome-wide association analyses with MR, this study provides a systematic framework for identifying genetically supported susceptibility genes associated with broad-spectrum glomerular dysfunction. Our analytical pipeline combines complementary approaches—including sCCA, FUSION, FOCUS, SMR, and sCCA-ACAT—to jointly model cross-tissue regulatory effects and refine potential candidate genes. Compared with conventional single-tissue analyses, this strategy improves detection sensitivity for regulatory signals operating across diverse biological contexts, thereby revealing associations that may remain obscured in traditional GWAS or tissue-restricted analyses.

Using this integrated framework, we initially identified 21 candidate genes associated with broad-spectrum glomerular dysfunction across multiple tissues. After rigorous validation using MR and sensitivity analyses, ten genes—*HSD17B8*, *TCF19*, *AGER*, *CYP21A2*, *PRRT1*, *C6orf48*, *LST1*, *HLA-DRB1*, *CSNK2B*, and *MICB*—remained robustly supported as putative susceptibility genes. Among the ten identified candidates, five genes—specifically *HSD17B8*, *PRRT1*, *C6orf48*, *LST1*, and *CSNK2B*—exhibited no prior evidence of direct association with GD according to our systematic search of PubMed, GWAS Catalog [43], Open Targets [44], and ClinVar [45], thereby representing novel susceptibility loci. Notably, five of the MR-validated genes—*AGER*, *CSNK2B*, *CYP21A2*, *HLA-DRB1* and *MICB*—encode proteins with druggable potential.

Five MR-supported genes—*TCF19*, *AGER*, *CYP21A2*, *HLA-DRB1*, and *MICB*—have been previously implicated in GD or related phenotypes, thereby reinforcing the credibility and reproducibility of our analytical strategy. *HLA-DRB1* has been reported to exhibit elevated expression in crescentic IgAN and serves as a primary genetic risk locus for PLA2R-related MN. Highlighting its fundamental role in the shared immunopathogenesis of diverse glomerular disorders [46,47]. Existing evidence suggests that *MICB* potentially mediates intrarenal immune activation through natural killer and T cells and has been associated with renal inflammatory injury and transplant rejection [48,49,50]. *AGER* has been associated with endothelial injury and mesangial expansion in diverse glomerular disorders, including DKD and FSGS, and has also been linked to MN [4,51]. Our prioritization of *CYP21A2* as a susceptibility gene for GD underscores the intricate and potentially dual roles of steroid metabolism in renal disease. While existing literature highlights its protective anti-inflammatory capacity in the specific context of IgAN, our cross-tissue evidence suggests that *CYP21A2* may harbor pathogenic properties in extra-renal or alternative tissue contexts, which collectively contribute to the genetic liability of GD [52]. In addition, the *TCF19* risk allele rs7750641-T has been associated with idiopathic MN and prioritized as a regulatory eGene at the 6p21.33 locus [43]. Our findings suggest that *TCF19* may contribute to shared glomerular pathogenesis through non-HLA-dependent pathways [53]. Our MR analysis further revealed a striking tissue-specific divergence in the genetically predicted effects of *TCF19* underlying this locus. Notably, increased *TCF19* expression in visceral adipose tissue was associated with an elevated risk of GD (OR = 1.47), whereas it exhibited a consistent protective effect across a broad range of other contexts, including the central nervous system and immune organs. These findings lead us to hypothesize a potential dual-pathway mechanism: in the metabolic microenvironment of the omentum, *TCF19* may drive the production of pro-inflammatory mediators, contributing to systemic microinflammation that exacerbates glomerular injury. Conversely, in non-adipose tissues, its primary role is postulated to involve maintaining cellular proteostasis and enhancing the resilience of glomerular cells against inflammatory insults.

Our findings further reinforce the central role of immunogenetic mechanisms in GD pathogenesis. Among the newly implicated genes, *LST1* emerged as a potential immunoregulatory factor. Although its role in GD has not been previously reported, *LST1* has been associated with immune regulation in several disease contexts, including the immune microenvironment of type 2 diabetes-related clear cell renal cell carcinoma and susceptibility to lupus nephritis [54,55]. Functional enrichment analyses further indicated that *LST1*, together with *AGER*, *HLA-DRB1*, and *CSNK2B*, participates in pathways related to lymphocyte proliferation and leukocyte activation. These genetic associations provide a basis to suggest glomerular inflammatory responses. Interestingly, *LST1* and *C6orf48* exhibited bidirectional associations across different tissues, which may reflect their functional plasticity within distinct biological microenvironments. For instance, the risk-increasing effect of *LST1* observed in the whole blood (OR = 3.06) provides a genetic basis to hypothesize a potential role in amplifying systemic inflammatory responses or leukocyte activation. Conversely, its protective association in cultured fibroblasts (OR = 0.85) potentially points to an involvement in maintaining cellular quiescence or promoting tissue repair within the structural framework, which warrants further experimental validation. Such tissue-specific pleiotropy underscores the complexity of the systemic-renal axis, where the net pathological impact of a gene depends on the balance of its regulatory roles across diverse tissues. *C6orf48*, located within the MHC class III region, has also been associated with chronic inflammatory diseases such as systemic lupus erythematosus and psoriatic arthritis [43,56]. The observation that several prioritized GD susceptibility genes (e.g., *HLA-DRB1*, *LST1*, and *C6orf48*) are heavily shared with other systemic inflammatory and autoimmune conditions, such as multiple sclerosis and lupus nephritis, is particularly noteworthy. This shared genetic architecture provides a genetic basis to hypothesize that GD pathogenesis is not strictly confined to the localized renal environment but rather shares fundamental, systemic immunological pathways with broader autoimmune disorders. Comparing these cross-disease mechanisms provides valuable insights: therapeutic strategies proven effective in these related systemic conditions—such as those modulating antigen presentation or mucosal immunity—may hold significant potential for drug repurposing in the treatment of GD.

In addition to immune mechanisms, our findings implicate metabolic and endocrine regulatory pathways in GD susceptibility. *HSD17B8* has been associated with mouse models of polycystic kidney disease and several cancers, indicating broader roles in cellular proliferation and metabolic regulation [57,58,59]. Enrichment analysis indicated that *HSD17B8* and *CYP21A2* participate in steroid hormone biosynthesis and lipid metabolism pathways, and PPI analysis revealed that HSD17B8 clusters with CYP21A2 and SRD5A family members. These findings suggest a hypothesis that *HSD17B8* could potentially influence glomerular homeostasis indirectly by modulating hormonal balance and mitochondrial fatty-acid metabolism, potentially contributing to the maintenance of the metabolic microenvironment that supports podocyte stability and cellular energy regulation.

Beyond immune and metabolic mechanisms, our results identify another module of cell-intrinsic regulatory genes that may influence GD susceptibility through modulation of the intracellular signaling network. Notably, *CSNK2B* exhibited a protective association (OR = 0.63), suggesting that its genetically supported higher expression is linked to reduced GD risk. Previous studies have implicated *CSNK2B* in neurodevelopment and erythropoiesis, including roles in cell proliferation [60,61,62,63]. As the regulatory subunit of protein kinase CK2, *CSNK2B* is known to be essential for the precise modulation of the PI3K/AKT/mTOR signaling network. In the context of the glomerulus, our findings suggest that robust *CSNK2B* expression may be critical for preserving the survival of intrinsic cells and maintaining cellular homeostasis under pathological stress. By promoting cellular resilience, *CSNK2B* is proposed to potentially assist in preventing the structural degradation and glomerulosclerosis typical of GD progression, based on our prioritized genetic evidence. Similarly, *PRRT1* showed a protective association (OR = 0.50), with increased expression in fibroblasts correlating with reduced GD risk. Although primarily studied in neuronal signaling, emerging evidence suggests broader roles in apoptosis, autophagy, and transmembrane signaling [64,65]. These observations lead to the hypothesis that *PRRT1* might contribute to maintaining fibroblast quiescence and extracellular matrix homeostasis within renal tissues. Together, *PRRT1* and *CSNK2B* may represent complementary components of a cell-intrinsic regulatory axis. Within this theoretical framework, *PRRT1* is proposed to contribute to structural stability and fibroblast quiescence, whereas *CSNK2B* enhances cellular resilience and survival through the modulation of intracellular signaling pathways. This interplay underscores the critical role of specific regulatory circuits in maintaining cellular quiescence, survival, and homeostatic balance, all of which are key determinants of GD susceptibility.

Building upon these mechanistic insights, identifying genetically supported targets provides a robust roadmap for precision intervention, though clinical translation must rigorously account for organ-specific pathophysiology. For instance, although AGER is formally classified as a Tier 3 target according to existing genomic frameworks, recent clinical developments highlight its rapidly evolving therapeutic potential. As RAGE serves as a critical co-receptor for suPAR—a key driver of glomerular injury—the progression of its antagonist Azeliragon (PF-4494700) into Phase 2/3 trials (NCT05815485) for acute kidney injury underscores its potential to suppress severe glomerular inflammation [66,67]. Conversely, while oncology-focused antibodies like CLN-619 (NCT05117476) aim to prevent MICB shedding, the genetically predicted pathogenic risk identified in our analysis suggests that GD treatment should instead prioritize competitive blockade of the NKG2D-MICB axis [68]. For HLA-DRB1 (a Tier 1 target), the FDA-approved glatiramer acetate theoretically interrupts antigen presentation; however, its clinical failure in lupus nephritis emphasizes that localized renal delivery may be essential to overcome the limitations of systemic immunomodulation [69].

The tissue-specific dynamics of CSNK2B and CYP21A2 further highlight the complexity of pharmacological translation in GD. Given the aforementioned protective role of CSNK2B in maintaining mucosal and cellular homeostasis, systemic CK2 inhibitors like Silmitasertib (CX-4945) could be counterproductive by disrupting barrier integrity [70]. Therapeutic strategies are proposed to instead focus on site-specific modulation or localized activation. Similarly, while congenital adrenal hyperplasia requires gene replacement to restore CYP21A2 function (e.g., BBP-631, NCT04783181), its potential pathogenic contribution to GD liability in extra-renal contexts suggests that the development of targeted inhibitors might be necessary to mitigate disease risk [71]. Ultimately, synergizing these genetic insights with existing pharmacological pipelines reinforces the rationale and highlights viable avenues for precision therapeutics in GD.

The utilization of the FinnGen N14_GLOMERULAR composite endpoint provides a cross-disease, systemic perspective for elucidating the genetic susceptibility to GD through a shared polygenic architecture. By identifying convergent pathogenic nodes—such as *HLA-DRB1*, which is implicated in both IgAN and MN, and *AGER*, linked to endothelial injury across the GD spectrum—our study highlights common immunometabolic dysregulation pathways that transcend traditional clinical boundaries. This approach is valuable given that many individual GD subtypes are often underpowered in GWAS; a composite endpoint thus maximizes the potential to detect core susceptibility factors governing the final common pathways of glomerular damage. Moreover, the inherent phenotypic heterogeneity of the cohort offers a rational biological explanation for the context-dependent functions of susceptibility genes. Despite the robustness of this integrative framework, interpreting these findings requires a balanced consideration of its limitations. While the aggregated phenotype facilitates the identification of a common denominator, it inherently risks masking or diluting genetic signals unique to specific subtypes [7]. While the identified genes represent potential candidates for a broad spectrum of glomerular dysfunction, their specific effect sizes and pathogenic roles may vary across distinct clinical entities. Furthermore, although potential horizontal pleiotropy and genetic signal distortion were heavily mitigated through rigorous sensitivity analyses (HEIDI, MR-Egger, and weighted median methods), the possibility of residual confounding cannot be entirely eliminated [24].

Additionally, the lack of an independent replication dataset remains a notable limitation, primarily due to the current scarcity of large-scale GWAS summary statistics matching the integrated phenotype of FinnGen cohort. Without replication in an external cohort, the findings of this study should be interpreted as an exploratory framework. Consequently, validation in independent, biopsy-proven cohorts for specific subtypes is still required to definitively confirm these candidate genes and further refine the genetic architecture of these distinct diseases. Furthermore, GTEx v8’s lack of pediatric data limits insights into early-onset GD [26]. Regarding our druggability analysis, it is important to note that while the MR framework genetically mimics the lifelong effects of target modulation, it cannot predict the long-term clinical response to actual pharmacological treatments. Complex temporal dynamics—such as drug resistance, delayed toxicities, and pharmacokinetics over prolonged administration—cannot be captured by static genetic data and remain a limitation to be addressed in future longitudinal clinical trials. Furthermore, our study relies on computational predictions without direct in vitro or in vivo experimental validation. While the integrated TWAS/MR framework is highly effective for rapidly screening candidates, our findings primarily offer predictive insights and cross-validation for identifying potential therapeutic targets in glomerular diseases. Lastly, there is currently a lack of large-scale targeted mutation clinical cohorts for immediate cross-validation. Thus, while our integrated pipeline provides statistically consistent prioritization, the precise biological roles of the newly prioritized candidate genes within the complex glomerular microenvironment remain speculative. Consequently, this study serves as an exploratory foundational roadmap, providing high-confidence hypotheses that warrant further experimental exploration to accurately characterize their causal mechanisms and therapeutic potential [6].

Future efforts should validate these genes in subtype-specific GD cohorts and diverse populations for generalizability [3]. Experimental studies, such as CRISPR editing in podocytes or mesangial cells, could clarify the roles of *HSD17B8* and *CSNK2B* [2]. Integrating single-cell RNA-seq with TWAS may refine cell-specific patterns [19]. Drug repurposing screens targeting *AGER* and *CYP21A2* could accelerate therapy development, leveraging their druggability (Table 1) [72].

## 5. Conclusions

This exploratory cross-tissue TWAS and MR study prioritized ten candidate susceptibility genes—*HSD17B8*, *TCF19*, *AGER*, *CYP21A2*, *PRRT1*, *C6orf48*, *LST1*, *HLA-DRB1*, *CSNK2B*, and *MICB*—associated with a broad spectrum of glomerular dysfunction (FinnGen N14_GLOMERULAR endpoint). These genes highlight immunogenetic (*HLA-DRB1*, *MICB*, *LST1*, etc.), metabolic (*HSD17B8* and *CYP21A2*) and cell-intrinsic regulatory (*CSNK2B* and *PRRT1*) pathways as key components of shared GD pathogenesis. Notably, *HSD17B8*, *PRRT1*, *C6orf48*, *LST1*, and *CSNK2B* represent novel candidates, offering new insights into GD’s molecular basis. The druggability of *AGER*, *CSNK2B*, *CYP21A2*, *HLA-DRB1* and *MICB* supports their potential as therapeutic targets, aligning with existing immunomodulatory and steroid-based treatments [6]. These findings advance our understanding of the shared genetic architecture across glomerular disorders and pave the way for precision medicine approaches. Future studies should validate these genes in subtype-specific cohorts and explore their mechanistic roles through functional experiments to guide targeted therapeutic development.

## Figures and Tables

**Table 1:** The druggability analysis of MR-validated genes.

Gene	Gene_id	Gene Name/Protein Product	Druggability Tier	Target Type	Drugs
*AGER*	ENSG00000204305	Advanced glycosylation end product receptor	3A	Clinical trial	PF-4494700
*C6orf48* (*SNHG32*)	ENSG00000204387	Small nucleolar RNA host gene 32	/ *	/	/
*CSNK2B*	ENSG00000204435	Casein kinase 2 beta	/	Clinical trial	Silmitasertib
*CYP21A2*	ENSG00000231852	Steroid 21-hydroxylase	3B	Clinical trial	BBP-631
*HLA-DRB1*	ENSG00000196126	MHC class II antigen DRB1*1	1	Successful	Glatiramer acetate
*HSD17B8*	ENSG00000204228	Hydroxysteroid 17-beta dehydrogenase 8	/	/	/
*LST1*	ENSG00000204482	Leukocyte specific transcript 1	/	/	/
*MICB*	ENSG00000204516	MHC class I polypeptide-related sequence B	/	Clinical trial	CLN-619
*PRRT1*	ENSG00000204314	Proline rich transmembrane protein 1	/	/	/
*TCF19*	ENSG00000137310	Transcription factor 19	/	/	/

* The slash symbol (“/”) indicates that data is unavailable. Specifically, in the “Druggability Tier” column, it denotes that a tier classification was not assigned by Finan et al. [42]; in the “Target Type” and “Drugs” columns, it signifies that no candidate drugs targeting the respective gene were retrieved. This applies to all “/” symbols throughout the table.

## Data Availability

Original data generated and analyzed during this study are included in the published article or the data repositories listed in the acknowledgments.

## Supplementary Materials

The following supporting information can be downloaded at: https://www.mdpi.com/article/10.3390/biomedicines14051072/s1, Table S1. Candidate genes associated with GD identified through sCCA; Table S2. sCCA results for cross-tissue gene expression patterns in GD; Table S3. FUSION identified 216 significant genes associated with GD; Table S4. FUSION identified 68 genes that were significant in the cross-tissue sCCA analysis; Table S5. COJO analysis of TWAS signals for glomerular disease; Table S6. MAGMA gene-based test identified 119 significant genes associated with GD; Table S7. The results of batch SMR analysis; Table S8. FOCUS fine-mapping results of gene-trait associations; Table S9. Multi-tissue Mendelian randomization analysis results using GTEx V8 data; Table S10. The functional pathway analysis related to 10 positive genes. Figure S1. Heatmap of FUSION−logFDR values for GD-associated genes across tissues. This visualization shows 216 genes with significant associations (FDR < 0.05) in at least one tissue. Higher values (red) indicate stronger statistical significance. Data presented in two panels for clarity. Figure S2. MAGMA gene-set, gene-level, and tissue enrichment analyses. (A) Functional enrichment and gene-level associations. The bar chart presents the functional enrichment results from the MAGMA gene-set analysis (GSA), revealing a significant enrichment in major histocompatibility complex (MHC) Class II pathways. This is complemented by a Manhattan plot illustrating the negative log-transformed *p*-values of individual genes, which highlights prominent gene-level signals at the *HLA-DQA1*, *PRRT1*, *TNXB*, *CLIC1*, *MSH5*, and *CYP21A2* loci. (B) Tissue enrichment analysis. The bar plot demonstrates the significance of enrichment across various tissue types based on MAGMA analysis. For both bar charts, the horizontal dashed line defines the threshold for statistical significance, and the color gradient of the bars corresponds to the *p*-value scale indicated in the right-hand legend. Figure S3. Functional enrichment analysis of susceptibility genes. The bar chart illustrates the functional enrichment results for the 10 susceptibility genes, performed using the Metascape platform. The analysis identifies two primary biological clusters and several specific molecular pathways. The most significant enrichment is observed in the regulation of lymphocyte proliferation (GO:0050670), which is primarily driven by *AGER*, *HLA-DRB1*, *LST1*, and *CSNK2B*. The x-axis displays the negative log-transformed *p*-values, representing the magnitude of statistical significance for each enriched Gene Ontology (GO) term, which also includes the regulation of hormone levels (GO:0010817).

## Abbreviations

The following abbreviations are used in this manuscript:

GD	glomerular diseases
CKD	chronic kidney disease
ESRD	end-stage renal disease
IgAN	IgA nephropathy
FSGS	focal segmental glomerulosclerosis
MN	membranous nephropathy
SNPs	single-nucleotide polymorphisms
LD	linkage disequilibrium
eQTL	expression quantitative trait loci
AD	Alzheimer’s disease
DKD	diabetic kidney disease
COJO	conditional and joint
GeneMANIA	Gene Multiple Association Network Integration Algorithm
EBI	European Bioinformatics Institute
PCA	principal components analysis
ACAT	Aggregate Cauchy Association Test
BLUP	best linear unbiased prediction
BSLMM	Bayesian sparse linear mixed model
LASSO	least absolute shrinkage and selection operator
FDR	false discovery rate
HEIDI	heterogeneity in dependent instruments
PIP	posterior inclusion probabilities
CI	confidence intervals
IVs	instrumental variables
PPI	protein–protein interaction
TTD	Therapeutic Target Database
GSA	gene-set analysis
MHC	major histocompatibility complex
OR	odds ratios
GO	Gene Ontology
