# Supplementary material for: A Cross-Tissue Transcriptome-Wide Association Study Identifies Novel Susceptibility Genes for Glomerular Diseases
Source: Biomedicines. 2026 May 8;14(5):1072. doi: 10.3390/biomedicines14051072 (PMC13203945; doi:10.3390/biomedicines14051072)
Supplement: Supplementary file 1 [file biomedicines-14-01072-s001.zip › Supplementary Files/Supplementary Figure S2.pdf]

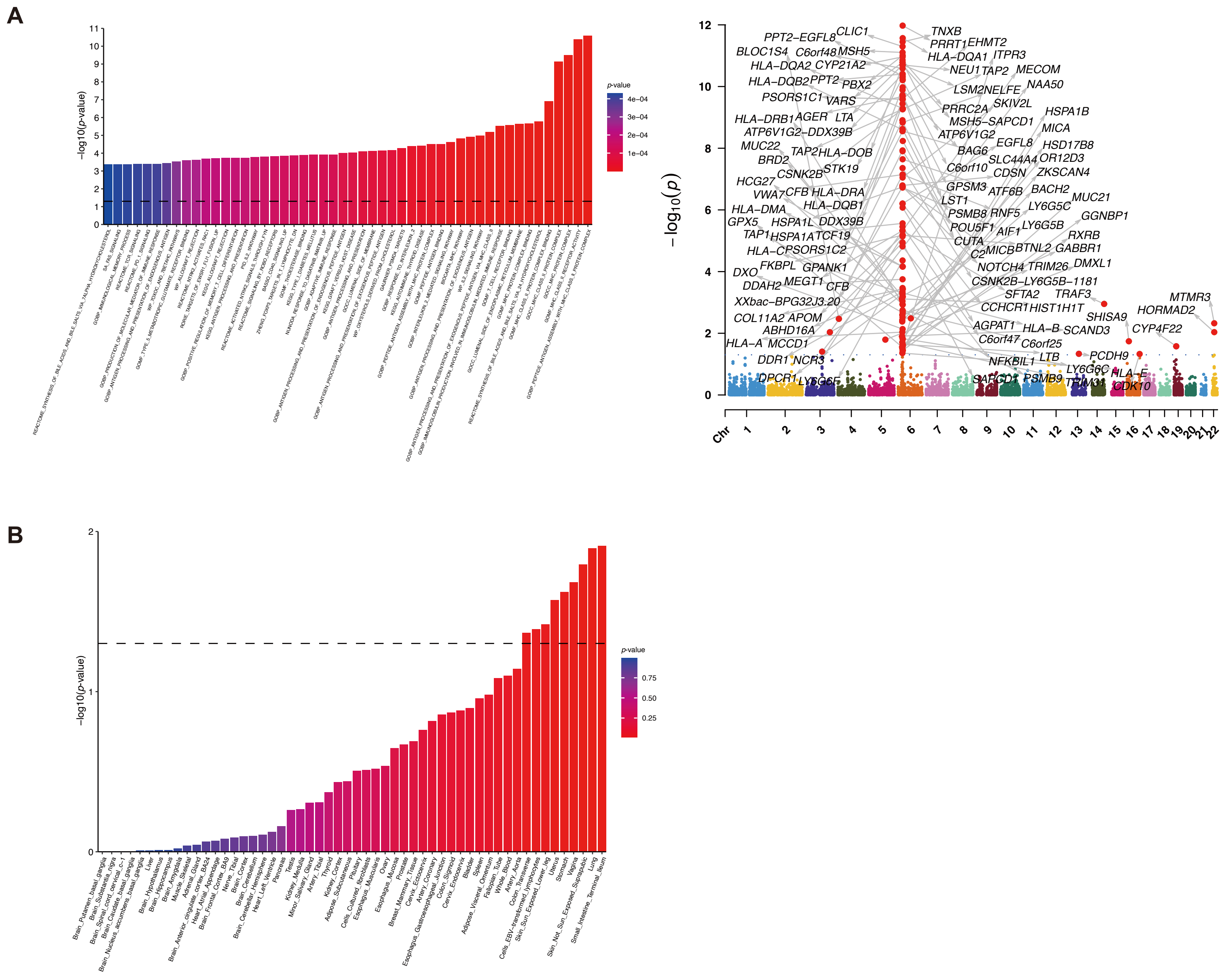

Supplementary Figure S2. MAGMA gene-set, gene-level, and tissue enrichment analyses. (A) Functional enrichment and gene-level associations. The bar chart presents the functional enrichment results from the MAGMA gene-set analysis (GSA), revealing a significant enrichment in major histocompatibility complex (MHC) Class II pathways. This is complemented by a Manhattan plot illustrating the negative log-transformed P-values of individual genes, which highlights prominent gene-level signals at the *HLA-DQA1, PRRT1, TNXB, CLIC1, MSH5*, and *CYP21A2* loci. (B) Tissue enrichment analysis. The bar plot demonstrates the significance of enrichment across various tissue types based on MAGMA analysis. For both bar charts, the horizontal dashed line defines the threshold for statistical significance, and the color gradient of the bars corresponds to the P-value scale indicated in the right-hand legend.
