# Supplementary material for: A Cross-Tissue Transcriptome-Wide Association Study Identifies Novel Susceptibility Genes for Glomerular Diseases
Source: Biomedicines. 2026 May 8;14(5):1072. doi: 10.3390/biomedicines14051072 (PMC13203945; doi:10.3390/biomedicines14051072)
Supplement: Supplementary file 1 [file biomedicines-14-01072-s001.zip › Supplementary Files/Supplementary Figure S3.pdf]

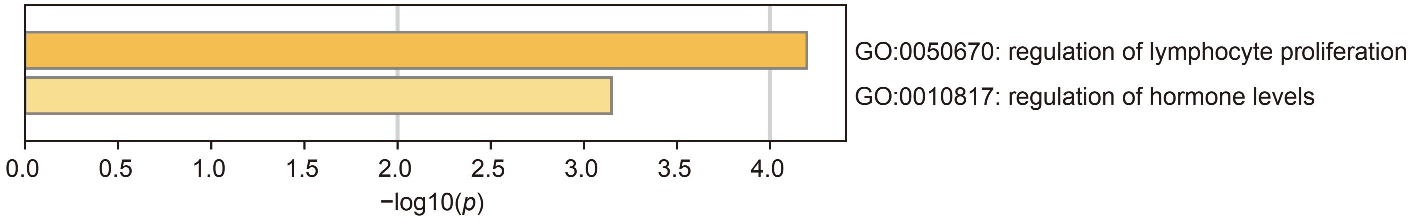

Supplementary Figure S3. Functional enrichment analysis of susceptibility genes. The bar chart illustrates the functional enrichment results for the 10 susceptibility genes, performed using the Metascape platform. The analysis identifies two primary biological clusters and several specific molecular pathways. The most significant enrichment is observed in the regulation of lymphocyte proliferation (GO:0050670), which is primarily driven by *AGER, HLA-DRB1, LST1,* and *CSNK2B*. The x-axis displays the negative log-transformed *p*-values, representing the magnitude of statistical significance for each enriched Gene Ontology (GO) term, which also includes the regulation of hormone levels (GO:0010817).
