# Supplementary material for: A Cross-Tissue Transcriptome-Wide Association Study Identifies Novel Susceptibility Genes for Glomerular Diseases
Source: Biomedicines. 2026 May 8;14(5):1072. doi: 10.3390/biomedicines14051072 (PMC13203945; doi:10.3390/biomedicines14051072)
Supplement: Supplementary file 1 [file biomedicines-14-01072-s001.zip › Supplementary Files/Supplementary Figure S1.pdf]

Heatmap of FUSION -logFDR values

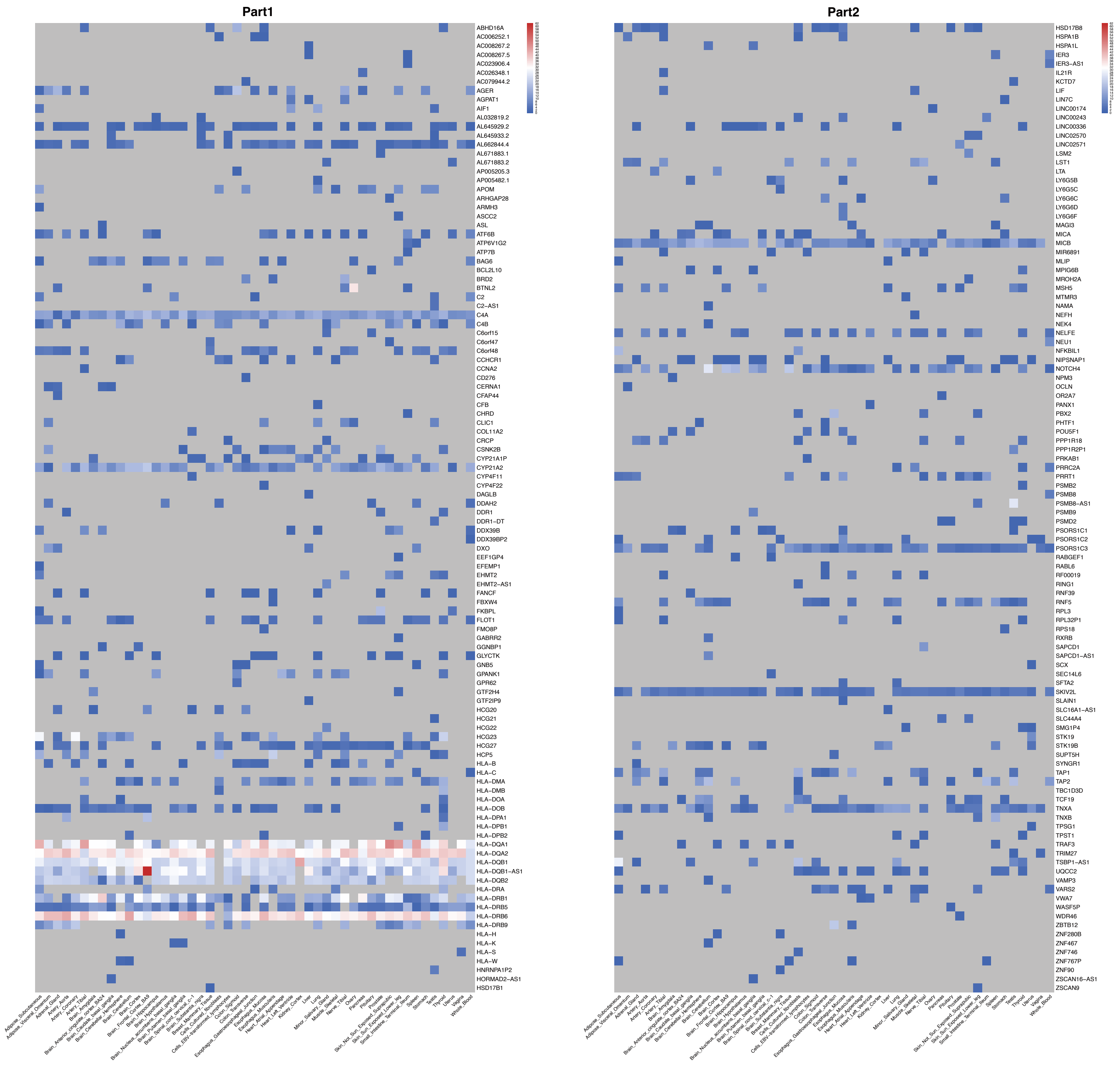

Supplementary Figure S1. Heatmap of FUSION -logFDR values for GD-associated genes across tissues. This visualization shows 216 genes with significant associations (FDR<0.05) in at least one tissue. Higher values (red) indicate stronger statistical significance. Data presented in two panels for clarity.
